# Diagnostic value of 5 miRNAs combined detection for breast cancer

**DOI:** 10.3389/fgene.2024.1482927

**Published:** 2024-11-25

**Authors:** Yubo Jing, Xinzhu Huang, Yiyang Wang, Junyi Wang, Yongxiang Li, Dlraba Yelihamu, Chenming Guo

**Affiliations:** Department of Breast Surgery, Center of Digestive and Vascular, The First Affiliated Hospital of Xinjiang Medical University, Urumqi, China

**Keywords:** breast cancer, microRNA, diagnosis, biomarker, bioinformatics

## Abstract

**Background:**

Breast cancer (BC) is the prevailing malignant tumor, with its prevalence and death rate steadily rising over time. BC often does not show obvious symptoms in its early stages and is difficult to distinguish from benign breast disease. We aimed to find a distinct group of miRNAs utilizing serum as a non-invasive biomarker for early BC diagnosis.

**Methods:**

Herein, we mainly include the screening stage, testing stage, and verification stage. In the screening stage, 8 miRNAs associated with BC were selected and analyzed via literature reading, and the expression of the above miRNAs in BC was further verified by bioinformatics and included in the research analysis. In the testing phase, quantitative reverse transcription polymerase chain reaction (qRT-PCR) was deployed to select the five miRNAs with the most significant expression differences in 15 BC patients and 15 benign breast controls to proceed to the next stage. In a subsequent validation phase, the five miRNAs obtained from serum samples from an additional 75 BC patients and 50 benign control patients were evaluated using RT-qPCR. The diagnostic capacity, specificity, and sensitivity of candidate miRNAs were estimated with the receiver operating characteristic (ROC) curve and area under the curve (AUC). Finally, the optimal diagnostic combination model with high sensitivity and strong specificity was constructed by using the above 5 miRNAs.

**Results:**

The BC patients reported a significant decline in mir-10b-5p, mir-133a-3p, mir-195-5p, and mir-155-3p levels in serum levels contrasted with those in benign controls. Additionally, BC patients experienced elevated mir-195-3p levels than in benign controls. We implemented ROC analysis to evaluate its diagnostic capacity for BC. We demonstrated that all five miRNAs had robust diagnostic capability, with an AUC above 0.8. We developed a conclusive diagnostic combination model consisting of these 5 miRNAs in order to enhance the diagnosis accuracy. This model demonstrated a high diagnostic value, as shown by an AUC of 0.948.

**Conclusion:**

The serum biomarker panels composed of five miRNAs identified in this study (mir-10b-5p, mir-133a-3p, mir-195-5p, mir-195-3p, and mir-155-3p) provide hope for early, non-invasive, and accurate diagnosis of BC.

## 1 Introduction

Breast Cancer (BC), a very prevalent and aggressive malignancy, seriously endangers women’s health and lives (Xu and Ma, 2020). Based on the statistics of the International Agency for Research on Cancer (IARC) in 2022, BC is the second-highest worldwide occurrence rate, surpassed only by lung cancer. There are nearly 2.3 million new instances and over 660,000 new deaths attributed to BC (Bray et al., 2024). The choice of treatment and prognosis of BC patients are closely related to early detection (Murobushi Ozawa et al., 2020). Over the last several years, the survival rate of BC patients has shown significant improvement due to ongoing advancements in screening and treatment techniques. However, the overall 5-year survival rate for patients in the late stages of the disease remains at a mere 20% (Bray et al., 2018; Harbeck et al., 2019). Early diagnosis of BC disease, further guidance of treatment, and improvement of prognosis are the key tasks in BC research at present. The commonly used auxiliary examination methods in clinical practice include breast ultrasound, molybdenum target, and tissue biopsy (Liu et al., 2022), among which tissue biopsy is currently the preferred method for obtaining tumor molecular information (Wu and Chu, 2022). In view of the limitations of existing early screening methods, new screening approaches are required to be developed and applied in clinical practice.

MicroRNA (miRNA) is a single non-coding RNA sequence with a length of 20–22 nucleotides that is present in several organisms encompassing humans, animals, plants, and other bodies and participates in RNA-mediated gene silencing. With people’s understanding of miRNA, studies have found that abnormal expression of miRNA genes will cause dysfunction and lead to diseases, especially cancer-related diseases (Gebert and MacRae, 2019), which has high reference value in distinguishing normal tissues and tumor subtypes. At the same time, due to their relatively simple molecular structure, their high tissue specificity and stability, and their ease of quantification and amplification, miRNAs are promising potential biomarkers.

Abundant evidence has shown the significant involvement of miRNA in developing several malignancies, including clear cell renal cell carcinoma (Shi et al., 2021), oral squamous cell carcinoma (Ran et al., 2024), and prostate cancer (Enokida et al., 2016). Research has shown that miRNA has an essential involvement in the biological processes of cancer cells, encompassing proliferation, differentiation, apoptosis, and invasion. It has emerged as a significant element in controlling cancer onset and progression (Khan et al., 2019). The issue of specific miRNAs as markers of tumor diagnosis and prognosis has been gradually paid attention by more and more scholars, which provides a new idea for the development of cancer diagnosis and treatment.

Current research results have confirmed that the miRNA expression could be linked to the occurrence and prognosis of BC (Aggarwal et al., 2020), but the impact of many miRNAs on its development is still unknown. We aimed to construct a specific set of miRNA combination models to explore their value in the diagnosis of BC and help timely identification of BC patients.

## 2 Materials and methods

### 2.1 Subjects and ethical statement

Herein, 90 patients with BC and 65 volunteers who served as benign controls were incorporated. The recruited patients were all patients who were admitted to the Department of Breast Surgery of the First Affiliated Hospital of Xinjiang Medical University from March 2023 to March 2024. All patients with BC were confirmed by preoperative puncture or postoperative histopathology. None of the patients included in this study had undergone any clinical treatment prior to the collection of serum specimens. The enrolled patients were all primary BC without metastases or other cancers. The TNM system used for staging classification and HER-2 status in all patients was diagnosed by assessment of histopathological parameters. None of the benign control volunteers had any history of acute or chronic diseases or other tumors. This investigation was examined and granted acceptance by the Ethics Committee of the First Affiliated Hospital of Xinjiang Medical University. The circumstances of the research were elucidated to all individuals, and each participant had perused and endorsed an informed consent form. In addition, the collection of serum samples and other research procedures strictly aligned with the relevant regulations of the Medical Ethics Committee of our hospital.

### 2.2 Collection and processing of blood samples

The following collection and sampling procedures were used to obtain serum specimens from patients and controls: Following admission, a sample of 5–10 mL of circulating blood in the morning while fasting was provided from all participants. The sterile tube containing without anticoagulant was implemented to take blood and then centrifuged at 3,000r at ambient temperature for 10 min. The supernatant was moved to a microcentrifuge tube and kept in a refrigerator at −80°C for future investigations.

### 2.3 Research design

The study was conducted in four stages. First, we searched for and screened 8 different miRNAs significantly related to BC expression as candidate biomarkers via a large number of literature reviews, and bioinformatics was used to further verify the differential expression of the above miRNAs in breast cancer. Second, additional testing and validation of these potential biomarkers were conducted. During the testing phase, the comparative levels of potential miRNAs in the serum were assessed with quantitative reverse transcription polymerase chain reaction (RT-qPCR) in 15 BC patients and 15 benign controls, selecting the top 5 miRNAs that showed the most significant differences at this stage for further study. Subsequently, the number of participants and replicated the aforementioned procedures were raised using serum specimens from 75 BC patients and 50 benign control patients. The aim was to confirm the findings from the prior phase. The expression levels and diagnostic potential of selected miRNAs were verified, and the diagnostic combination model consisted of candidate miRNAs to improve diagnostic sensitivity and specificity. Finally, the potential biological functions of candidate miRNAs related to BC generation were explored with bioinformatics analysis.

### 2.4 RNA extraction and detection

miRNA was extracted by miRNA extraction kit (DP503, Tiangen)on mycycler instrument (T100, Bio-Rad, USA) under reverse transcription conditions of 70°C for 10 min, 4°C for 2 min, 42°C for 60 min, 70°C for 10 min. The ABI QuantStudio 5 was used to conduct qPCR, which included denaturing at 95°C for 10 min, followed by 40 cycles of denaturing at 95°C for 15 s and annealing and extension at 60°C for 1 min. Every specimen was subjected to three technical duplicates. Each transcript concentration was afterward normalized to cel-mir-39 (*caenorhabditis elegans* microRNA-39) and mRNA level with the 2^−ΔΔCT^ technique for analysis (Livak and Schmittgen 2001) (Supplementary Table S1). The comparisons were conducted using the two-way ANOVA analysis in GraphPad Prism software (Version 8.0, San Diego, CA).

### 2.5 Bioinformatics analysis

The expression levels of five miRNAs in breast cancer tumors were analyzed using the Cancer Genome Atlas (TCGA) database (https://portal.gdc.cancer.gov/) (Filippi and Mocanu, 2023). Related target genes in miRDB, mirTarBase, and TargetScan databases in five miRNAs were analyzed using miRWalk (http://mirwalk.umm.uni-heidelberg.de/), and Venn diagrams were drawn to visualize the results. The main biological functions of miRNAs, their prediction pathways, and linked functions were ascertained with Gene Ontology (GO) and the Kyoto Encyclopedia of Genes and Genomes (KEGG).

### 2.6 Statistical analysis

The data in this research was analyzed using SPSS software. The Shapiro-Wilk normality test is implemented to evaluate the adherence of continuous variables to a normal distribution. Categorical variables are shown as percentages for various groups, whereas continuous variables that follow a normal distribution are reported as the mean ± standard deviation. A P-value below 0.05 was deemed significant. The miRNA levels in BC and benign control specimens were compared using a t-test for continuous variables, while a χ^2^ test was used for categorical variables. Each potential miRNA diagnostic efficacy was assessed with the receiver operating characteristic (ROC) curve and the area under the ROC curve (AUC). The specificity, sensitivity, and overall diagnostic capabilities of each miRNA were assessed using these measures. In addition, we calculated the Jorden index (J = sensitivity + specificity −1) to ascertain the ideal integration of miRNAs that produces the greatest levels of sensitivity and specificity for diagnostic purposes.

## 3 Results

### 3.1 Confirmation of candidate miRNAs

**FIGURE 1:**
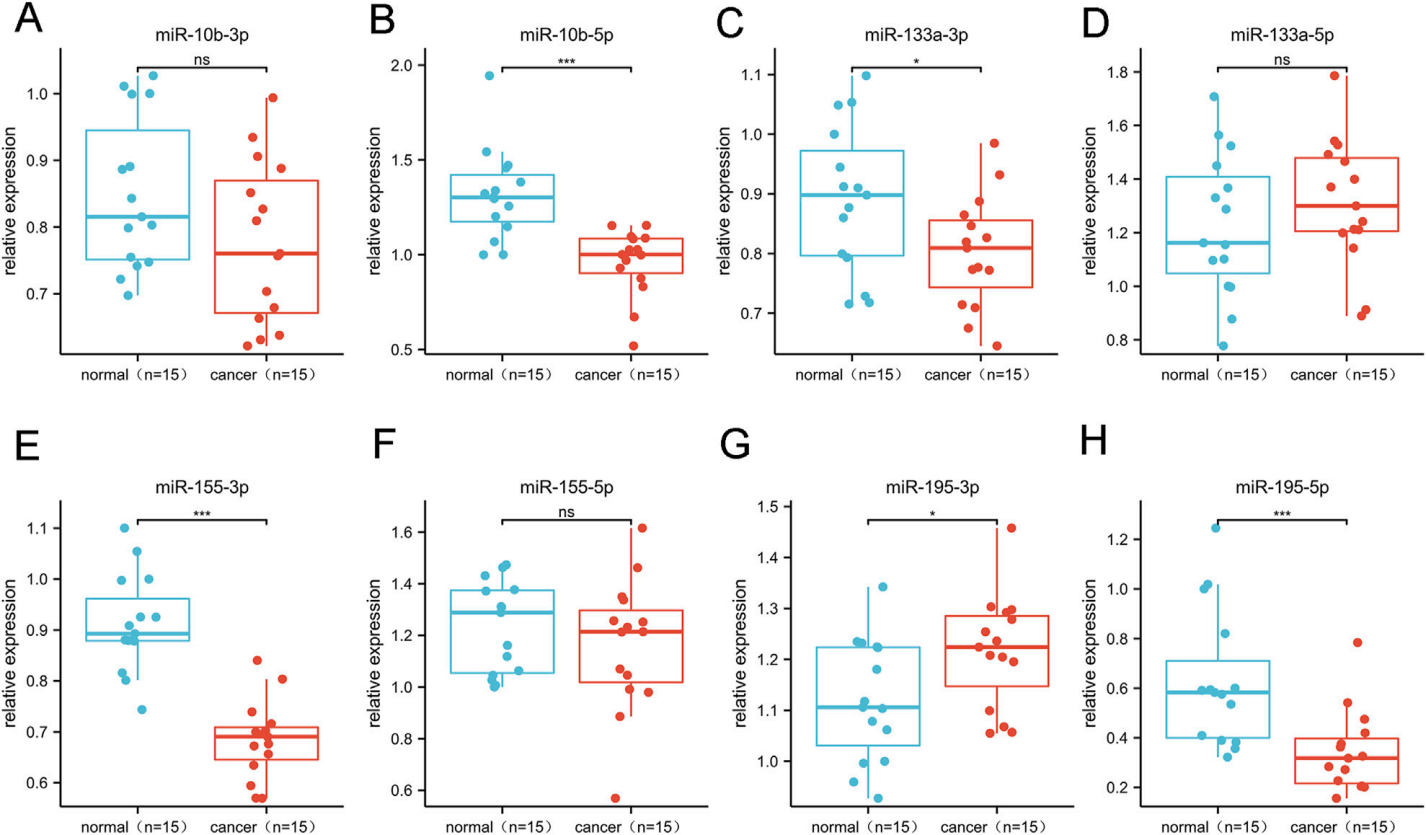
Differential expression of 8 candidate miRNAs. This phase comprised 15 BC and 15 control serum specimens.

We selected and refined the selection of BC-related candidate miRNAs via a large number of literature reviews to reviewing the results of previous experiments, selected BC-related miRNAs as candidate miRNAs for subsequent research in this study (Table 1). The miRNA precursors produce two complementary functional mature miRNAs, generally processed from the 5 and 3′end arm of the precursor, respectively named “-5p” and “-3p”, which target different sites (Alsharafi et al., 2015). Therefore, a total of 8 complementary miRNAs were incorporated in this investigation. We used bioinformatics tools to further validate the differential expression of the above miRNAs in BC. Through the analysis of the expression content of target miRNAs in 1109 invasive breast cancer tissues (Tumor) and 113 normal tissues in the TCGA database, it was found that the target miRNAs (miR-10b-5p, miR-10b-3p, miR-133a-5p, miR-133a-3p, miR-155-5p, miR-155-3p, miR-195-5p, miR-195-3p) was expressed differently in the above unpaired tissues (p< 0.05; Supplementary Figure S1). In addition, similar results were obtained in 112 pairs of paired breast cancer tissues, with significantly lower levels of target miRNAs expression in the tumor group than in the normal group (p< 0.05; Supplementary Figure S2). In addition, in our study, we utilized the TCGA database to validate the diagnostic value of the above-mentioned miRNAs in breast cancer patients (Supplementary Figure S3). The results showed that mir-10b-5p, mir-133a-3p, mir-155-3p, mir-195-3p and mir-195-5p all showed high diagnostic efficiency (AUC>0.9). We conducted an RT-qPCR analysis on a random subgroup of 15 BC patients and 15 individuals with benign controls to evaluate eight specific miRNA levels in the serum (Figures 1A–H). Our study found significant differences in the mir-10b-5p (Figure 1B), mir-133a-3p (Figure 1C), mir-155-3p (Figure 1E), mir-195- 3p (Figure 1G), and mir-195-5p (Figure 1H) expression levels.

**TABLE 1:** Diagnostic value of different mirnas in patients with breast cancer.

Target miRNA	Sample type	Case group	Control group	AUC	Sensitivity	Specificity	References
miRNA-10	Serum	61	48	0.75	0.79	0.77	Dwedar et al. (2021)
	Serum	99	40	0.77	0.60	0.93	Mohamed et al. (2022)
miRNA-133	plasma	76	27	0.79	—	—	Shen et al. (2014)
miRNA-155	Serum	99	40	0.94	0.87	0.90	Mohamed et al. (2022)
	plasma	41	32	0.70	0.78	0.75	Itani et al. (2021)
	Serum	36	36	0.89	0.78	0.89	Hosseini Mojahed et al. (2020)
	Serum	158	107	0.82	0.83	0.80	Huang et al. (2018)
	Serum	40	30	0.99	0.97	0.94	Swellam et al. (2019)
	Serum	99	21	0.75	1.00	0.51	Han et al. (2017)
	Serum	152	40	0.78	0.85	0.70	Eichelser et al. (2013)
	Serum	103	55	0.80	0.65	0.82	Sun et al. (2012)
miRNA-195	Serum	210	102	0.859	0.69	0.89	Zhao et al. (2014)

### 3.2 Clinical and demographic features of investigation subjects

We incorporated 155 subjects, including 90 BC patients and 65 volunteers with benign breast lesions. Table 2 displays the clinical and demographic features of the participants throughout the testing and validation stages, with a mean age of 49.9 ± 7.5 in the BC group and 44.5 ± 9.8 in the benign control group. Participants did not change significantly in age or menopausal status. The average age of participants enrolled in the study after the enlarged sample was 51.5 ± 7.0, and the average age of the benign control group was 40.4 ± 9.5. They have significant differences in age and menopausal status, which may be due to hormonal effects. Reproductive factors such as age and menopausal status are particularly closely related to the occurrence of BC (Shah et al., 2022). We further analyzed whether the expression of these five miRNAs was associated with clinicopathological features in breast cancer patients. In our study, we found that the expression of miR-10b-5p was significantly associated with Her-2, T stage, N stage, different stages of cancer, and PAM50 subtype (Supplementary Table S2). The expression of miR-133a-3p was found to be significantly correlated with Her-2, T stage, and different stages of cancer (Supplementary Table S3). However, when patients were stratified by pathological TNM stage, PR status, and HER2 status, no significant differences in mir-195-3p, mir-195-5p, and mir-155-3p expression were observed (Supplementary Tables S4–S6). According to these outcomes, additional analysis particularly prioritized these five miRNAs. After univariate analysis, we found that all five miRNAs included in the analysis were significantly linked to the occurrence of BC (Table 3). Due to the strong collinearity of the molecules, it is impossible to conduct a multi-factor analysis.

**TABLE 2:** General demographic characteristics of study participants were included.

	Testing phase (n = 30)			Validation phase (n = 125)		
	BC(n = 15)	Control (n = 15)	P-Value	BC (n = 75)	Control (n = 50)	P-Value
Age, year	49.9 ± 7.5	44.5 ± 9.8	0.10	51.5 ± 7.0	40.4 ± 9.5	<0.05
Postmenopausal status, n (%)						
Premenopausal	9 (60)	11 (73.3)	0.70	28 (37.3)	37 (74.0)	<0.05
Postmenopausal	6 (40)	4 (26.7)		47 (62.7)	13 (26.0)	

**TABLE 3:** Single factor analysis of occurrence of 5 miRNAs and BC.

Characteristics	Total(N)	Univariate analysis	
		OR (95% CI)	P-Value
miR-10b-5p	125	0.019 (0.004–0.090)	<0.001
miR-133a-3p	125	0.000 (0.000–0.001)	<0.001
miR-155-3p	125	0.000 (0.000–0.003)	<0.001
miR-195-5p	125	0.231 (0.131–0.408)	<0.001
miR-195-3p	125	9.052 (2.242–36.551)	0.002

### 3.3 Diagnostic value of 5 candidate miRNAs

**FIGURE 2:**
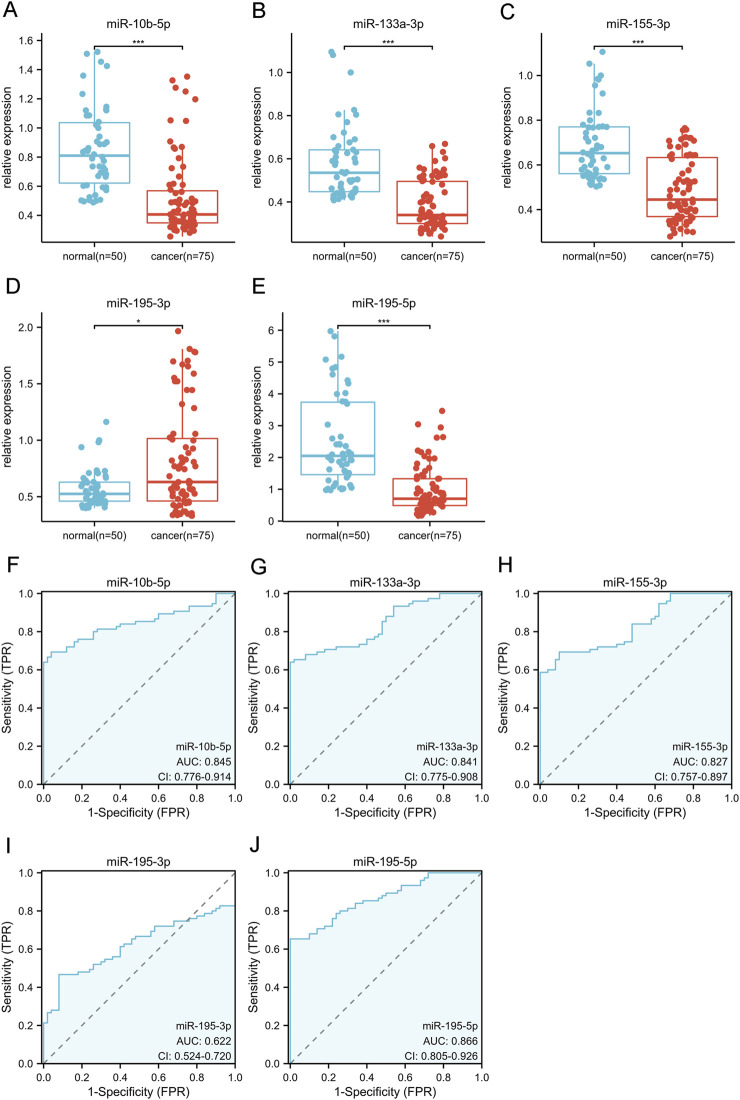
The validation phase of five selected miRNAs involves the use of Relative expression counting and receiver operating characteristic curve (ROC) analyses. This phase comprised 75 BC and 50 control serum specimens. The area under the curve (AUC) of the five miRNAs were: 0.845 [95% CI: 0.776–0.914, *p* = 0.014 **(F)**] for miR-10b-5p **(A)**, 0.841 [95% CI: 0.775–0.908, *p* < 0.001 **(G)**] for miR-133a-3p **(B)**, 0.827 [95% CI:0.757–0.897, *p* < 0.001 **(H)**] for miR-155-3p **(C)**, 0.622 [95% CI:0.524–0.720, p< 0.001 **(I)**] for miR-195-3p **(D)**, and 0.866 [95% CI: 0.805–0.926, *p* = 0.007 **(J)**] for miR-195-5p **(E)**, respectively. *p < 0.05, **p < 0.01,***p < 0.001.

We increased the number of samples incorporated in the investigation to verify the efficacy of five particular miRNAs as serum biomarkers for BC screening. We comprised 75 BC patients and 50 controls with benign conditions. The RT-qPCR method was deployed to assess the relative serum levels of 5 miRNAs. The BC patients demonstrated significant elevation in mir-10b-5p, mir-133a-3p, mir-155-3p, and mir-195-5p levels contrasted with benign controls (Figures 2A–E). The BC experienced an elevated mir-195-3p expression level contrasted with benign controls (Figure 2D). These five miRNAs’ diagnostic effectiveness was evaluated with ROC curve examination. The AUC for each miRNA is shown in Figures 2F–J. The Jorden index was next used to determine the appropriate threshold, specificity, and sensitivity for diagnosing BC using the five miRNAs (Table 4). The ROC curve analysis revealed that mir-10b-5p, mir-133a-3p, mir-195-5p, mir-195-3p, and mir-155-3p had promising diagnostic capabilities for BC, with respective AUC values of 0.85, 0.84, 0.87, 0.62, and 0.83.

**TABLE 4:** Optimal threshold, specificity and sensitivity of 5 miRNAs for the diagnosis of BC.

Target miRNA	Cut-off value	AUC	Sensitivity	Specificity	Accuracy
miR-10b-5p	0.50	0.85	0.69	0.96	0.80
miR-133a-3p	0.41	0.84	0.64	1.00	0.78
miR-155-3p	0.54	0.83	0.70	0.90	0.78
miR-195-3p	0.74	0.62	0.47	0.92	0.65
miR-195-5p	0.96	0.87	0.65	1.00	0.79

### 3.4 Construction of optimal diagnosis model

**FIGURE 3:**
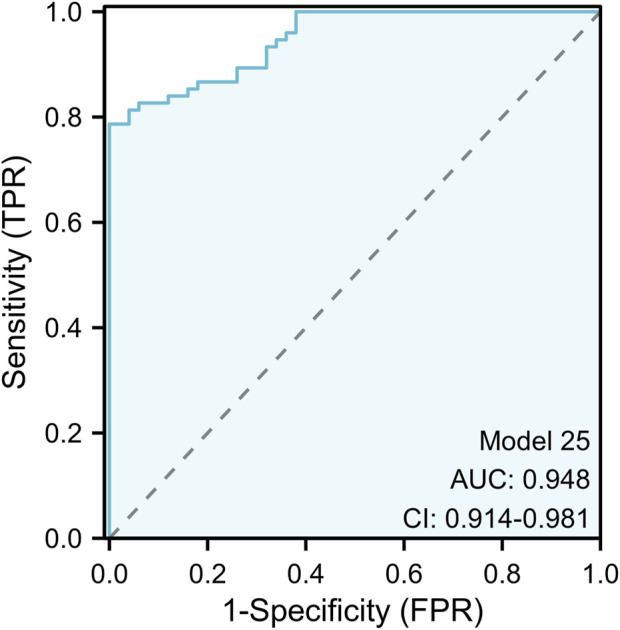
The ROC curve assessment for the five-miRNA panel. The five-miRNA panel consisted of mir-10b-5p, mir-133a-3p, mir-155-3p, mir-195-5p, and mir-195-3p and the AUC of five-miRNA panel was 0.948 (95%CI: 0.914 to 0.981; *p* < 0.001; sensitivity = 0.79; specificity = 1.00).

In the experimental stage, we found that mir-10b-5p, mir-133a-3p, mir-195-5p, mir-195-3p, and mir-155-3p had good diagnostic ability for BC. Hence, we conducted an investigation to estimate whether combinations of many miRNAs exhibited greater diagnostic efficacy for BC and determined the optimal integration for BC diagnosis from all possible miRNA combinations (Table 5). Our study found that almost all of the models constructed except for the three diagnostic models, 15, 17, and 21, had extremely high specificity (>0.90), suggesting that all models could accurately identify the occurrence of BC. In addition, our study found that models 4, 19, and 24 all had high diagnostic values (AUC >0.90), possibly because they jointly included miR-10b-5p and miR-195-5p, which were the best diagnostic in a single miRNA. Model 25 was composed of 5 miRNAs. This model had extremely high diagnostic capability, and the ROC curve showed an AUC of 0.948 (95% CI: 0.914 − −0.981; Specificity = 100%, Sensitivity = 79%). The formula of the model is: Logit (*p* = 0.757) + (46.427 * miR - 10 b - 5 P) + 29.354 * (miR-133a-3P) + (20.170 * miR-155-3 P) + (3.144 * miR - 195-3 P) + (17.138 * miR - 195-5 P). The outcome is superior to each separate miRNA, as seen in Figure 3.

**TABLE 5:** Specificity and sensitivity of all combined models of 5 miRNAs.

No.	Target miRNA	AUC	Sensitivity	Specificity	Accuracy
1	miR-10b-5p + miR-133a-3p	0.86	0.67	1.00	0.80
2	miR-10b-5p + miR-155-3p	0.82	0.57	1.00	0.74
3	miR-10b-5p + miR-195-3p	0.86	0.72	1.00	0.83
4	miR-10b-5p + miR-195-5p	0.92	0.76	0.94	0.82
5	miR-133a-3p + miR-155-3p	0.89	0.73	1.00	0.84
6	miR-133a-3p + miR-195-5p	0.86	0.65	1.00	0.79
7	miR-133a-3p + miR-195-3p	0.86	0.65	1.00	0.79
8	miR-195-5p + miR-195-3p	0.89	0.65	1.00	0.79
9	miR-155-3p + miR-195-3p	0.84	0.67	1.00	0.80
10	miR-155-3p + miR-195-5p	0.88	0.65	1.00	0.79
11	miR-133a-3p + miR-155-3p + miR-195-3p	0.88	0.72	1.00	0.83
12	miR-133a-3p + miR-155-3p + miR-195-5p	0.89	0.72	1.00	0.83
13	miR-155-3p + miR-195-5p + miR-195-3p	0.86	0.64	1.00	0.78
14	miR-10b-5p + miR-133a-3p + miR-155-3p	0.90	0.77	1.00	0.86
15	miR-10b-5p + miR-195-5p + miR-195-3p	0.94	0.85	0.88	0.86
16	miR-10b-5p + miR-155-3p + miR-195-3p	0.85	0.63	1.00	0.78
17	miR-10b-5p + miR-155-3p + miR-195-5p	0.92	0.95	0.78	0.88
18	miR-10b-5p + miR-133a-3p + miR-195-3p	0.89	0.64	1.00	0.78
19	miR-10b-5p + miR-133a-3p + miR-195-5p	0.92	0.72	1.00	0.83
20	miR-133a-3p + miR-155-3p + miR-195-5p + miR-195-3p	0.88	0.72	1.00	0.83
21	miR-10b-5p + miR-133a-3p + miR-155-3p + miR-195-3p	0.90	0.83	0.86	0.84
22	miR-10b-5p + miR-133a-3p + miR-155-3p + miR-195-5p	0.94	0.80	0.96	0.86
23	miR-10b-5p + miR-155-3p + miR-195-5p + miR-195-3p	0.93	0.73	0.98	0.83
24	miR-10b-5p + miR-133a-3p + miR-195-3p + miR-195-5p	0.93	0.73	1.00	0.84
25	miR-10b-5p + miR-133a-3p + miR-155-3p + miR-195-5p + miR-195-3p	0.95	0.79	1.00	0.87

### 3.5 Bioinformatics analysis of 5 miRNAs

**FIGURE 4:**
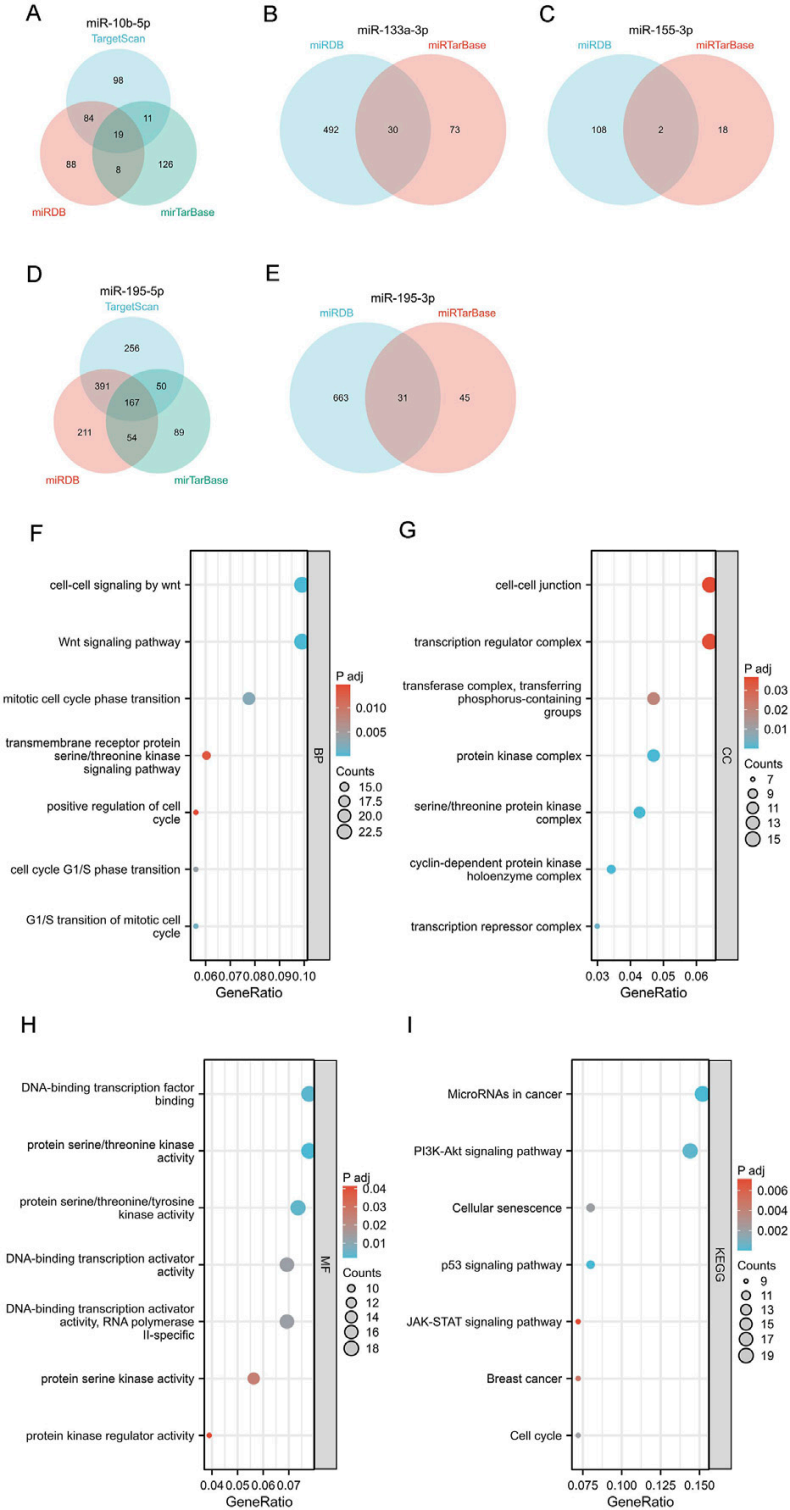
Target gene prediction, GO function annotation, and KEGG pathway enrichment analysis of 5 miRNAs by miRWalk3.0. **(A–E)** Target genes of mir-10b-5p, mir-133a-3p, mir-155-3p, mir-195-5p, and mir-195-3p. Biological process (BP) analysis **(F)**, cellular component (CC) analysis **(G)**, molecular function (MF) analysis **(H)**, and KEGG pathway enrichment analysis **(I)**.

We used miRNAWalk2.0 to explore and visualize the potential target genes of mir-10b-5p, mir-133a-3p, mir-155-3p, mir-195-5p, and mir-195-3p (Figures 4A–E). In addition, the potential target genes of the above 5 miRNAs were included in KEGG pathway enrichment analysis and GO function to analyze their potential functions further(Supplementary Table S7). The results indicate that the genes targeted by mir-10b-5p, mir-133a-3p, mir-195-5p, mir-195-3p, and mir-155-3p possess a significant function in the pathways and processes that are crucial for the genesis and progression of cancer. The GO analysis findings indicated that the aforementioned miRNAs mostly participated in biological processes such as WNT-mediated cell-cell signaling, WNT signaling pathway, and mitosis cell cycle phase transition (Figure 4F) and were also mainly involved in cell junction, transcriptional regulatory complex, transferase complex, and the transfer of phosphorus-containing groups and other cell components (Figure 4G). In addition, our study found that they are also involved in molecular functions encompassing DNA-binding transcription factor binding and protein serine/threonine kinase activity (Figure 4H). KEGG analysis results showed that the above five miRNAs play a role mainly through mediating microRNAs, PI3K-Akt signaling pathway, and cell senescence in cancer (Figure 4I). The target gene enrichment in these pathways indicates their possible involvement in controlling crucial cellular processes linked to the development of tumors.

## 4 Discussion

BC is one of the prevailing types of malignant tumors. In recent years, BC incidence and mortality have increased year by year, and the age of onset has shown a younger trend (Fan et al., 2014). BC usually does not show obvious symptoms in the early stages, is not easy to detect in the course of routine physical examination, and is difficult to distinguish from benign breast diseases. However, as the disease progresses, the tumor may become malignant and metastasize, leading to a poor prognosis. Hence, the early detection of BC poses a significant challenge. Biomarkers offer a beneficial role in facilitating the BC clinical diagnosis. Currently, some serum markers have been found to have a certain relationship with the onset of BC (Barzaman et al., 2020) and have been clinically verified. Recently, several non-coding RNAs have also been investigated for their potential involvement in assisting in the detection of BC. MiRNAs are short fragments of non-coding ribonucleic acid (ncRNAs) that exist in biological fluids. Due to their high tissue specificity and stability, miRNAs have been increasingly applied to distinguish normal tissues from tumor subtypes (Mishra et al., 2016). Prior investigations have reported that plasma miRNAs can be used as a biomarker to detect BC.

In this study, 8 miRNAs with diagnostic potential in BC were selected through an extensive literature review. Bioinformatics tools were used to further verify the differential expression of the above miRNAs in BC. Serums of 15 benign controls and 15 BC patients were examined to detect the diagnostic significance of these 8 miRNAs in BC. The findings of our study validate that the mir-10b-5p, mir-133a-3p, mir-195-5p, and mir-155-3p levels were significantly mitigated in comparison to the levels found in healthy individuals. Additionally, the mir-195-3p expression level was greater in BC individuals compared to healthy controls. The variations in expression indicate that miRNAs could have a role in the growth and advancement of BC. ROC curve analysis demonstrated that all five of the aforementioned miRNAs had significant diagnostic potential, indicating that these microRNAs show potential as non-invasive biomarkers for the early detection and diagnosis of BC. We then expanded the sample size to 75 BC patients and 50 benign control patients for further verification. Based on the above five high-quality miRNAs, we have successfully built the best diagnostic panel with high sensitivity and strong specificity. Prior investigations have explored the diagnostic performance of each of the 5 miRNAs mentioned above separately, but no studies have included the above 5 miRNAs together in the diagnostic tool. Our study found that the integrated panel demonstrates superior diagnostic accuracy and performance compared to each individual miRNA. Therefore, the above five miRNAs combined into a diagnostic combination panel could be regarded as a new biomarker for the BC diagnosis.

Both Mir-195-3P/5P belong to the mir-195 family and are contributing to the tumor’s occurrence and development. Zhao et al. (2014) showed that contrasted with the healthy control group, the serum miR-195 level in the early BC group was reduced by more than two times, revealing that miR-195 might be used as a biological marker for the early BC diagnosis. Our study reached a similar conclusion. In addition, our study also found that two complementary mature miRNAs of miR-195 have good diagnostic value. Out of the five individual miRNAs that were analyzed, mir-195-5p demonstrated the most effective diagnostic capabilities, with exceptional sensitivity and specificity (65% and 100%, respectively). Within the diagnostic panel we developed, mir-195-3p stands out as the only miRNA that exhibits overexpression, specifically in cases of BC. Prior research has shown a correlation between mir-195-3p and BC. Nevertheless, the mechanism by which mir-195-3p contributes to the development of BC has not yet been investigated. Mir-195-5p is regarded as a new predictive indicator for rectal cancer (Bayat et al., 2022). Cervical cancer reported a hindrance in mir-195-5p expression level compared to the control group. This decrease in expression may hinder the malignant advancement of cervical cancer, specifically targeting YAP1 (Liu et al., 2020). These studies provide data suggesting that miR-195 might serve as a biological marker for diagnosing BC.

Prior investigations have reported that the miR-10b family contributes to various tumorigenesis and metastasis processes. It has been proved that the serum miR-10b-5p expression level in early hepatocellular carcinoma (HCC) patients is significantly increased, and miR-10b-5p has a high diagnostic value (ROC = 0.934) (Cho et al., 2020). Furthermore, prior investigation from our research group has shown a significant connection between the miR-10b-5p expression and the molecular subtypes of early invasive ductal carcinoma (Guo et al., 2018). Additionally, subsequent investigations by our research group have revealed that high miR-10b-5p expression serves as a protective factor for patients with breast invasive ductal carcinoma, leading to reduced rates of recurrence and mortality. It might serve as a promising treatment target for individuals diagnosed with breast-invasive ductal carcinoma. In conjunction with this research, we posit that miR-10b-5p could operate as a new biomarker for identifying and anticipating BC prognosis in patients.

Mir-133a-3p has also been ascertained as a significant controller of HCC and could potentially operate as a non-invasive biomarker for diagnosing HCC patients (AUC = 0.67) (Liang et al., 2018). Furthermore, elevated expression of mir-133a-3p was found in non-small cell lung cancer (NSCLC) patients contrasted with the control group. Nevertheless, the accuracy of this diagnosis still has to be confirmed by further verification (Xue et al., 2020). Currently, there are insufficient investigations determining the connection between mir-133a-3p and the BC diagnosis. Our investigation is the initial to find that mir-133a-3p has a strong ability to distinguish BC from benign breast diseases (ACU = 0.84), and it is also the first study to include it in the diagnostic tool.

As a miRNA that regulates the core role of cancer, miR-155 has been found to be highly correlated with gynecological tumors (Fan et al., 2018), digestive system tumors (Nassar et al., 2021), and hematological system tumors (Zheng et al., 2019), and more and more proof demonstrates that its 3p chain possesses a certain contribution in these fields. Zhang et al. (2019) found that compared with the paired normal tissues, the miR-155-3p expression level in BC tissues was shown to be lower, which aligns with our study findings. The results of our investigation indicate that the miR-155-3p expression level could function as a biomarker for the early detection of BC, demonstrating a high degree of sensitivity, specificity, and accuracy.

Moreover, we performed bioinformatics analyses using miRNA Walk 2.0 to predict potential downstream target genes for these miRNAs. The KEGG pathway enrichment analysis indicated that these genes were mostly enriched in several cancer pathways, such as the Wnt, PI3K, and P53 signaling pathways. Prior research has shown that miR-10b-5p may expedite the process of glycometabolic reprogramming in gliomas by stimulating the PI3K/Akt pathway (Li et al., 2024). Moreover, mir-133a-3p has been confirmed to impact the progression of thyroid cancer (Xia and Jie, 2020) and prostate cancer (Tang et al., 2018) by participating in the PI3K/Akt pathway. Mir-195-5p is regarded as a possible therapeutic target for renal cell carcinoma because it modulates the WNT pathway in this kind of cancer (Chen et al., 2017). mir-195-3p, which belongs to the same family, is also believed to contribute to the proliferation, migration, and invasion of cancer cells in renal cell carcinoma (Jin et al., 2017). In addition, mir-195-3p has also been confirmed to participate in multiple cancer pathways in lung adenocarcinoma (Lao et al., 2022) and hepatocellular carcinoma (Mei et al., 2022), thereby affecting the occurrence and development of malignant tumors. Mir-155-3p, a crucial element in the cancer pathway, has been discovered to control the proliferation of colorectal cancers (Li et al., 2021). The dysregulation of mir-10b-5p, mir-133a-3p, mir-195-5p, mir-195-3p, and mir-155-3p in BC indicates that they could operate as valuable diagnostic and prognostic biomarkers for this condition. To summarize, our data provide more evidence of the function of miRNA in the development of BC.

MiRNA-based biomarkers provide several benefits, such as their resilience in serum samples, capacity to be detected non-invasively, and possibility for early diagnosis. In this study, five miRNAs with high correlation with BC were selected from clinical samples to draw a diagnostic model with higher diagnostic efficiency, overcoming the shortcomings of single miRNA diagnosis. A better distinction between the BC population and the breast benign disease population provides the possibility of early diagnosis of BC by combination model. At present, researchers in various fields are working to explore and confirm blood-based biomarkers. Some scholars have also found that some miRNAs can be implemented as potential diagnostic indicators for BC patients. However, some researchers believe that the sensitivity and specificity of a single marker assay are low, which is not enough to meet the clinical needs of diagnosing diseases, so some studies have confirmed that the combined analysis of several target miRNAs can find that the combined miRNAs have higher accuracy, sensitivity and AUC, and have a more convincing diagnostic basis. Elhelbawy NG et al. (Elhelbawy et al., 2021) reported that mir-148a and mir-30c had better diagnostic efficacy in BC than traditional CA 15-3 and CEA. Nevertheless, the current investigation possessed a limited sample size and the combined diagnostic efficacy of the two miRNAs was not explored, which may affect the reliability of their diagnostic value. Kumar et al. (2023) constructed a combined diagnostic model of three miRNAs for non-invasive diagnosis of triple-negative BC. However, the team has not yet validated it in other BC subtypes. Five kinds of miRNAs with individual diagnostic efficacy were included in our study. A diagnostic model composed of mir-10b-5p, mir-133a-3p, mir-195-5p, mir-195-3p, and mir-155-3p was developed as a diagnostic marker for BC, which is conducive to timely intervention and treatment decision-making. Improved patient management. Nevertheless, more validation studies involving bigger groups of individuals are necessary to verify the diagnostic accuracy and therapeutic usefulness of these miRNAs.

## 5 Conclusion

In this study, we preliminarily identified BC-associated serum miRNA markers and looked for 5 serum miRNAs that possess the capacity to be implemented in BC screening and early detection. Furthermore, we have effectively developed a diagnostic panel using these 5 miRNAs, which exhibits exceptional diagnostic performance, sensitivity, and specificity. This breakthrough offers a novel avenue for the timely detection of BC.

## Data Availability

The original contributions presented in the study are included in the article/Supplementary Material, further inquiries can be directed to the corresponding author.

## Supplementary material

SUPPLEMENTARY FIGURE S1 Differences in the expression of miRNAs in the TCGA database between unpaired breast cancer tissues and normal tissues.

SUPPLEMENTARY FIGURE S2 Differences in the expression of miRNAs in TCGA database between paired breast cancer tissues and normal tissues.

SUPPLEMENTARY FIGURE S3 The TCGA database was used to verify the diagnostic efficiency of the target miRNA in breast cancer.
