# Supplementary figures and images for: Diagnostic value of 5 miRNAs combined detection for breast cancer
Source: Front Genet. 2024 Nov 25;15:1482927. doi: 10.3389/fgene.2024.1482927 (PMC11625769; doi:10.3389/fgene.2024.1482927)

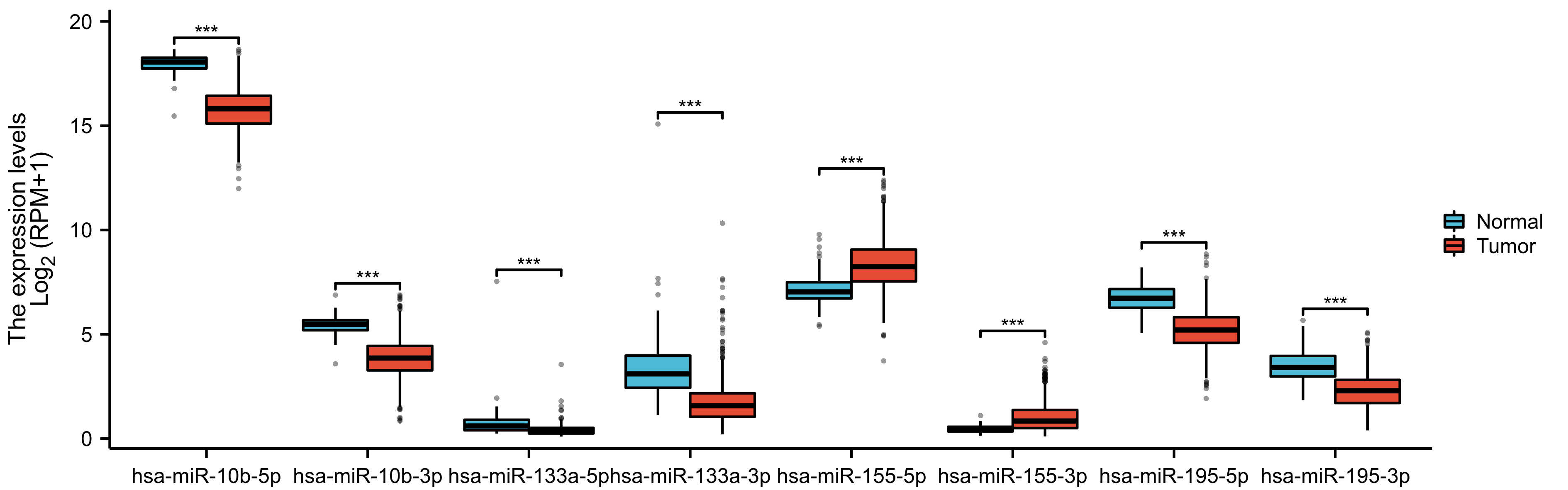

Supplement: Supplementary file 1 [file Image1.tiff]

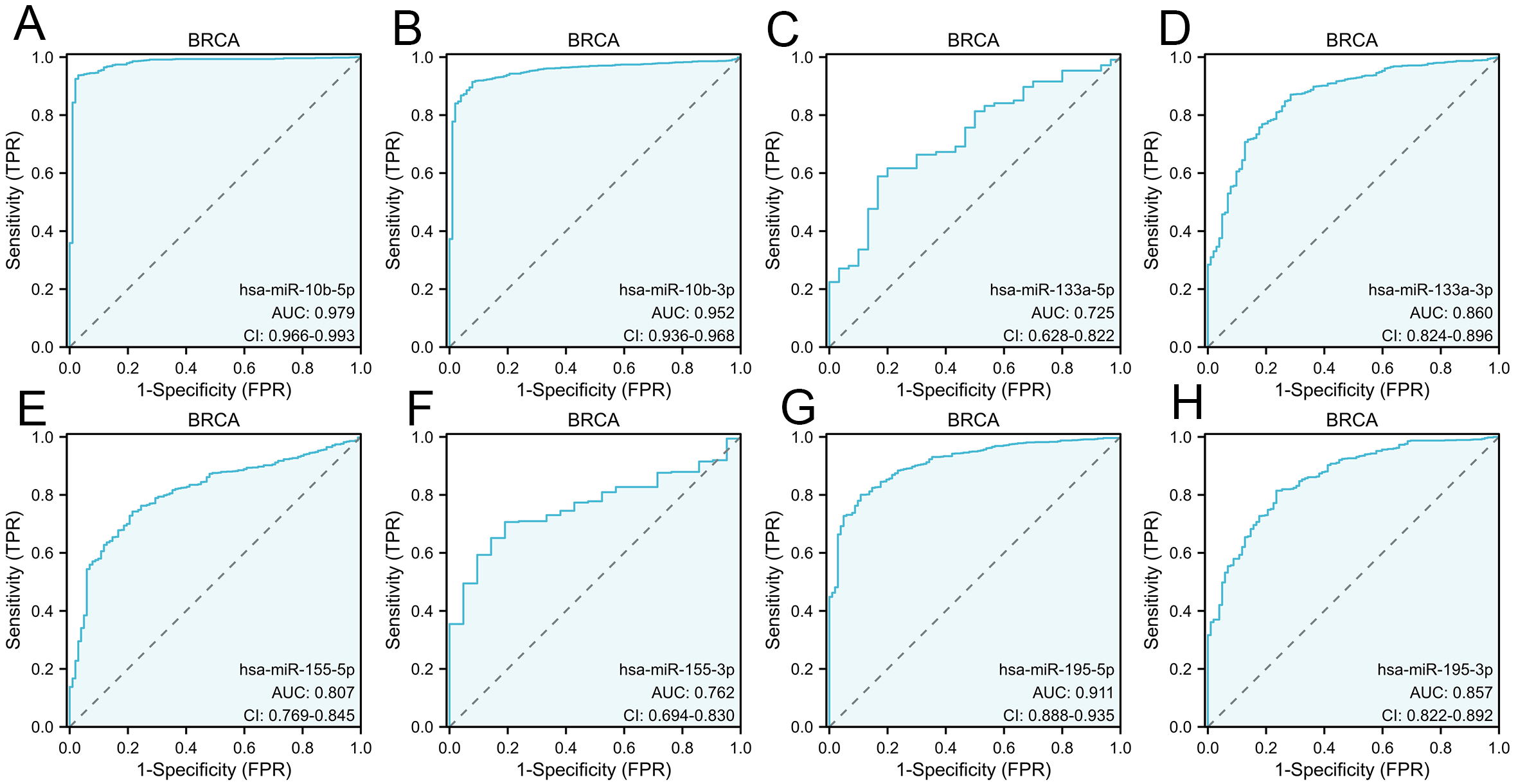

Supplement: Supplementary file 3 [file Image3.tif]

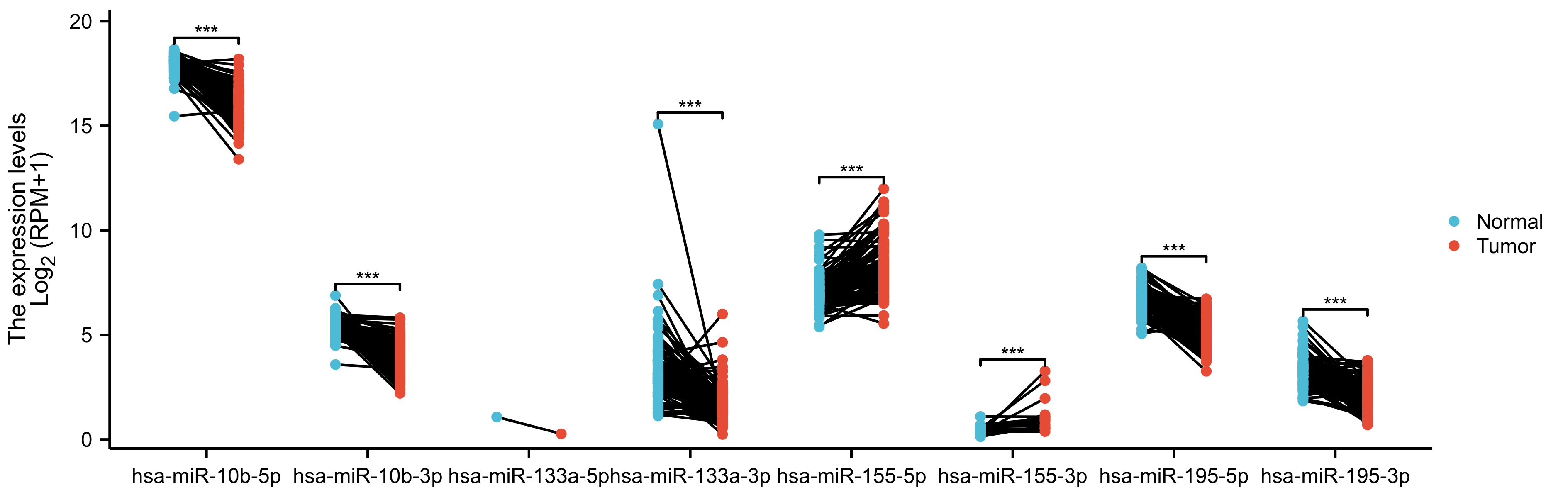

Supplement: Supplementary file 7 [file Image2.tiff]
