# Supplementary material for: Diagnostic value of 5 miRNAs combined detection for breast cancer
Source: Front Genet. 2024 Nov 25;15:1482927. doi: 10.3389/fgene.2024.1482927 (PMC11625769; doi:10.3389/fgene.2024.1482927)
Supplement: Supplementary file 2 [file DataSheet4.docx]

**Table S4.** Correlation between Mir-195-5p and clinicopathological features in breast cancerpatients from LinkedOmics database.

| Characteristics | Low expression of  miR-195-5p | High expression of  miR-195-5p | P value |
| --- | --- | --- | --- |
| n | 37 | 38 |  |
| Age, n (%) |  |  | 0.831 |
| ≤60 | 29 (38.7%) | 29 (38.7%) |  |
| ＞60 | 8 (10.7%) | 9 (12%) |  |
| Menopausal State, n (%) |  |  | 0.677 |
| Pre & Peri | 11 (14.7%) | 13 (17.3%) |  |
| Post | 26 (34.7%) | 25 (33.3%) |  |
| ER, n (%) |  |  | 0.119 |
| Positive | 31 (41.3%) | 26 (34.7%) |  |
| Negative | 6 (8%) | 12 (16%) |  |
| PR, n (%) |  |  | 0.117 |
| Positive | 26 (34.7%) | 20 (26.7%) |  |
| Negative | 11 (14.7%) | 18 (24%) |  |
| Her-2, n (%) |  |  | 0.843 |
| Positive | 28 (37.3%) | 28 (37.3%) |  |
| Negative | 9 (12%) | 10 (13.3%) |  |
| Pathologic T stage, n (%) |  |  | 0.336 |
| T1 | 15 (20%) | 13 (17.3%) |  |
| T2 | 19 (25.3%) | 16 (21.3%) |  |
| T3 | 1 (1.3%) | 3 (4%) |  |
| T4 | 2 (2.7%) | 6 (8%) |  |
| Pathologic N stage, n (%) |  |  | 0.389 |
| N0 | 18 (24%) | 20 (26.7%) |  |
| N1 | 16 (21.3%) | 11 (14.7%) |  |
| N2 | 1 (1.3%) | 4 (5.3%) |  |
| N3 | 2 (2.7%) | 3 (4%) |  |
| Pathologic M stage, n (%) |  |  | 0.130 |
| M0 | 37 (49.3%) | 34 (45.3%) |  |
| M1 | 0 (0%) | 4 (5.3%) |  |
| Pathologic stage, n (%) |  |  | 0.157 |
| Stage I | 13 (17.3%) | 11 (14.7%) |  |
| Stage II | 17 (22.7%) | 13 (17.3%) |  |
| Stage III | 7 (9.3%) | 10 (13.3%) |  |
| Stage IV | 0 (0%) | 4 (5.3%) |  |
| PAM50, n (%) |  |  | 0.061 |
| LumA | 5 (6.7%) | 10 (13.3%) |  |
| LumB | 15 (20%) | 7 (9.3%) |  |
| Her2 | 15 (20%) | 14 (18.7%) |  |
| Basal | 2 (2.7%) | 7 (9.3%) |  |
