# Supplementary material for: Diagnostic value of 5 miRNAs combined detection for breast cancer
Source: Front Genet. 2024 Nov 25;15:1482927. doi: 10.3389/fgene.2024.1482927 (PMC11625769; doi:10.3389/fgene.2024.1482927)
Supplement: Supplementary file 4 [file DataSheet3.docx]

**Table S3.** Correlation between Mir-195-3p and clinicopathological features in breast cancerpatients from LinkedOmics database.

| Characteristics | Low expression of  miR-195-3p | High expression of  miR-195-3p | P value |  |  |  |
| --- | --- | --- | --- | --- | --- | --- |
| n | 38 | 37 |  |  |  |  |
| Age, n (%) |  |  | 0.735 |  |  |  |
| ≤60 | 30 (40%) | 28 (37.3%) |  |  |  |  |
| ＞60 | 8 (10.7%) | 9 (12%) |  |  |  |  |
| Menopausal State, n (%) |  |  | 0.566 |  |  |  |
| Pre & Peri | 11 (14.7%) | 13 (17.3%) |  |  |  |  |
| Post | 27 (36%) | 24 (32%) |  |  |  |  |
| ER, n (%) |  |  | 0.634 |  |  |  |
| Positive | 28 (37.3%) | 29 (38.7%) |  |  |  |  |
| Negative | 10 (13.3%) | 8 (10.7%) |  |  |  |  |
| PR, n (%) |  |  | 0.742 |  |  |  |
| Positive | 24 (32%) | 22 (29.3%) |  |  |  |  |
| Negative | 14 (18.7%) | 15 (20%) |  |  |  |  |
| Her-2, n (%) |  |  | 0.739 |  |  |  |
| Positive | 29 (38.7%) | 27 (36%) |  |  |  |  |
| Negative | 9 (12%) | 10 (13.3%) |  |  |  |  |
| Pathologic T stage, n (%) |  |  | 0.679 |  |  |  |
| T1 | 14 (18.7%) | 14 (18.7%) |  |  |  |  |
| T2 | 18 (24%) | 17 (22.7%) |  |  |  |  |
| T3 | | 1 (1.3%) | 3 (4%) |  |  |  |
| T4 | 5 (6.7%) | 3 (4%) |  |  |  |  |
| Pathologic N stage, n (%) |  |  | 0.066 |  |  |  |
| N0 | 23 (30.7%) | 15 (20%) |  |  |  |  |
| N1 | 12 (16%) | 15 (20%) |  |  |  |  |
| N2 | 0 (0%) | 5 (6.7%) |  |  |  |  |
| N3 | 3 (4%) | 2 (2.7%) |  |  |  |  |
| Pathologic M stage, n (%) |  |  | 0.627 |  |  |  |
| M0 | 35 (46.7%) | 36 (48%) |  |  |  |  |
| M1 | 3 (4%) | 1 (1.3%) |  |  |  |  |
| Pathologic stage, n (%) |  |  | 0.393 |  |  |  |
| Stage I | | | 12 (16%) | 12 (16%) |  |  |
| Stage II | 17 (22.7%) | 13 (17.3%) |  |  |  |  |
| Stage III | 6 (8%) | 11 (14.7%) |  |  |  |  |
| Stage IV | 3 (4%) | 1 (1.3%) |  |  |  |  |
| PAM50, n (%) |  |  | 0.978 |  |  |  |
| LumA | 7 (9.3%) | 8 (10.7%) |  |  |  |  |
| LumB | 11 (14.7%) | 11 (14.7%) |  |  |  |  |
| Her2 | | | | 15 (20%) | 14 (18.7%) |  |
| Basal | 5 (6.7%) | 4 (5.3%) |  |  |  |  |
