# Supplementary material for: Diagnostic value of 5 miRNAs combined detection for breast cancer
Source: Front Genet. 2024 Nov 25;15:1482927. doi: 10.3389/fgene.2024.1482927 (PMC11625769; doi:10.3389/fgene.2024.1482927)
Supplement: Supplementary file 5 [file DataSheet5.docx]

**Table S5.** Correlation between Mir-155-3p and clinicopathological features in breast cancerpatients from LinkedOmics database.

| Characteristics | Low expression of  miR-155-3p | High expression of  miR-155-3p | P value |  |
| --- | --- | --- | --- | --- |
| n | 38 | 37 |  |  |
| Age, n (%) |  |  | 0.373 |  |
| ≤60 | 31 (41.3%) | 27 (36%) |  |  |
| ＞60 | 7 (9.3%) | 10 (13.3%) |  |  |
| Menopausal State, n (%) |  |  | 0.285 |  |
| Pre & Peri | 10 (13.3%) | 14 (18.7%) |  |  |
| Post | 28 (37.3%) | 23 (30.7%) |  |  |
| ER, n (%) |  |  | 0.948 |  |
| Positive | 29 (38.7%) | 28 (37.3%) |  |  |
| Negative | 9 (12%) | 9 (12%) |  |  |
| PR, n (%) |  |  | 0.535 |  |
| Positive | 22 (29.3%) | 24 (32%) |  |  |
| Negative | 16 (21.3%) | 13 (17.3%) |  |  |
| Her-2, n (%) |  |  | 0.388 |  |
| Positive | 30 (40%) | 26 (34.7%) |  |  |
| Negative | 8 (10.7%) | 11 (14.7%) |  |  |
| Pathologic T stage, n (%) |  |  | 0.883 |  |
| T1 | 15 (20%) | 13 (17.3%) |  |  |
| T2 | 18 (24%) | 17 (22.7%) |  |  |
| T3 | 2 (2.7%) | 2 (2.7%) |  |  |
| T4 | 3 (4%) | 5 (6.7%) |  |  |
| Pathologic N stage, n (%) |  |  | 0.104 |  |
| N0 | 22 (29.3%) | 16 (21.3%) |  |  |
| N1 | 13 (17.3%) | 14 (18.7%) |  |  |
| N2 | 0 (0%) | 5 (6.7%) |  |  |
| N3 | 3 (4%) | 2 (2.7%) |  |  |
| Pathologic M stage, n (%) |  |  | 0.627 |  |
| M0 | 35 (46.7%) | 36 (48%) |  |  |
| M1 | 3 (4%) | 1 (1.3%) |  |  |
| Pathologic stage, n (%) |  |  | 0.068 |  |
| Stage I | | 13 (17.3%) | 11 (14.7%) |  |
| Stage II | 18 (24%) | 12 (16%) |  |  |
| Stage III | 4 (5.3%) | 13 (17.3%) |  |  |
| Stage IV | 3 (4%) | 1 (1.3%) |  |  |
| PAM50, n (%) |  |  | 0.195 |  |
| LumA | 8 (10.7%) | 7 (9.3%) |  |  |
| LumB | 10 (13.3%) | 12 (16%) |  |  |
| Her2 | 18 (24%) | 11 (14.7%) |  |  |
| Basal | 2 (2.7%) | 7 (9.3%) |  |  |
