# Supplementary material for: Diagnostic value of 5 miRNAs combined detection for breast cancer
Source: Front Genet. 2024 Nov 25;15:1482927. doi: 10.3389/fgene.2024.1482927 (PMC11625769; doi:10.3389/fgene.2024.1482927)
Supplement: Supplementary file 8 [file DataSheet1.docx]

**Table S1.** Correlation between Mir-10b-5p and clinicopathological features in breast cancerpatients from LinkedOmics database.

| Characteristics | Low expression of  miR-10b-5p | High expression of  miR-10b-5p | P value |
| --- | --- | --- | --- |
| n | 38 | 37 |  |
| Age, n (%) |  |  | 0.188 |
| ≤60 | 27 (36%) | 31 (41.3%) |  |
| ＞60 | 11 (14.7%) | 6 (8%) |  |
| Menopausal State, n (%) |  |  | 0.118 |
| Pre & Peri | 9 (12%) | 15 (20%) |  |
| Post | 29 (38.7%) | 22 (29.3%) |  |
| ER, n (%) |  |  | 0.948 |
| Positive | 29 (38.7%) | 28 (37.3%) |  |
| Negative | 9 (12%) | 9 (12%) |  |
| PR, n (%) |  |  | 0.884 |
| Positive | 23 (30.7%) | 23 (30.7%) |  |
| Negative | 15 (20%) | 14 (18.7%) |  |
| Her-2, n (%) |  |  | 0.014 |
| Positive | 33 (44%) | 23 (30.7%) |  |
| Negative | 5 (6.7%) | 14 (18.7%) |  |
| Pathologic T stage, n (%) |  |  | < 0.001 |
| T1 | 23 (30.7%) | 5 (6.7%) |  |
| T2 | 11 (14.7%) | 24 (32%) |  |
| T3 | 1 (1.3%) | 3 (4%) |  |
| T4 | 3 (4%) | 5 (6.7%) |  |
| Pathologic N stage, n (%) |  |  | 0.004 |
| N0 | 27 (36%) | 11 (14.7%) |  |
| N1 | 8 (10.7%) | 19 (25.3%) |  |
| N2 | 1 (1.3%) | 4 (5.3%) |  |
| N3 | 2 (2.7%) | 3 (4%) |  |
| Pathologic M stage, n (%) |  |  | 0.588 |
| M0 | 37 (49.3%) | 34 (45.3%) |  |
| M1 | 1 (1.3%) | 3 (4%) |  |
| Pathologic stage, n (%) |  |  | < 0.001 |
| Stage I | 22 (29.3%) | 2 (2.7%) |  |
| Stage II | 11 (14.7%) | 19 (25.3%) |  |
| Stage III | 4 (5.3%) | 13 (17.3%) |  |
| Stage IV | 1 (1.3%) | 3 (4%) |  |
| PAM50, n (%) |  |  | 0.015 |
| LumA | 5 (6.7%) | 10 (13.3%) |  |
| LumB | 10 (13.3%) | 12 (16%) |  |
| Her2 | 21 (28%) | 8 (10.7%) |  |
| Basal | 2 (2.7%) | 7 (9.3%) |  |
